# Radiomic Analysis for Ki-67 Classification in Small Bowel Neuroendocrine Tumors

**DOI:** 10.3390/cancers18030463

**Published:** 2026-01-30

**Authors:** Filippo Checchin, Davide Malerba, Alessandro Gambella, Aurora Rita Puleri, Virginia Sambuceti, Alessandro Vanoli, Federica Grillo, Lorenzo Preda, Chandra Bortolotto

**Affiliations:** 1 Diagnostic Imaging and Radiotherapy Unit, Department of Clinical, Surgical, Diagnostic, and Pediatric Sciences, University of Pavia, 27100 Pavia, Italy; davide.malerba01@universitadipavia.it (D.M.); aurorarita.puleri01@universitadipavia.it (A.R.P.); lorenzo.preda@unipv.it (L.P.); chandra.bortolotto@unipv.it (C.B.); 2 Department of Surgical Sciences and Integrated Diagnostics (DISC), University of Genoa, 16132 Genoa, Italy; alessandro.gambella@unige.it (A.G.); federica.grillo@unige.it (F.G.); 3 Pathology Unit, IRCCS Azienda Ospedaliera Metropolitana, 16132 Genoa, Italy; 4 Department of Radiology, IRCCS Azienda Ospedaliera Metropolitana, 16132 Genoa, Italy; virginia.sambuceti@hsanmartino.it; 5 Department of Internal Medicine, University of Genoa, 16132 Genoa, Italy; 6 Unit of Anatomic Pathology, Department of Molecular Medicine, University of Pavia, 27100 Pavia, Italy; alessandro.vanoli@unipv.it; 7 Unit of Anatomic Pathology, IRCCS San Matteo Hospital Foundation, 27100 Pavia, Italy; 8 Radiology Institute, Fondazione IRCCS Policlinico San Matteo, 27100 Pavia, Italy

**Keywords:** neuroendocrine tumors, radiomics, Ki67

## Abstract

**Objective**: To analyze radiomic features extracted from CT images of small bowel neuroendocrine tumors and evaluate their association with Ki-67 expression. **Methods**: 128 small bowel NET primary and secondary lesions from 34 patients were analyzed. Manual segmentation of the lesions was conducted on portal-phase CT images using ITK-SNAP v. 4.0^®^, and 107 radiomic features were extracted using the PyRadiomics library. The lesions were categorized into two groups based on their Ki-67 index expression (≤1% and >1%). Correlation filtering reduced the set of 107 to 41 radiomic features. Inferential statistical analyses (*t*-test and Mann–Whitney U, following Shapiro–Wilk and Levene’s tests) identified 19 significant features (*p* < 0.05) that were predominantly texture related. A ranking procedure further reduced these to eight top-performing variables across multiple selection methods (Information Gain, Gini, ANOVA, χ^2^). Five supervised Machine Learning models (Logistic Regression, Support Vector Machine (SVM), K-Nearest Neighbors (KNN), XGBoost, and Random Forest) were trained and validated using 5-fold cross-validation. The evaluation metrics employed included AUC, accuracy, precision, recall, F1 score, and a confusion matrix. **Results**: Random Forest exhibited the best overall performance (AUC = 0.80; F1 score = 0.813; Recall = 0.847). The model’s low false negative rate (15.3%) suggests potential clinical utility in minimizing the risk of underestimating more aggressive lesions. **Conclusions**: Radiomics represents a promising frontier to identify patterns associated with histopathological markers. This study highlights its potential for non-invasive assessment of proliferative rate in small bowel neuroendocrine tumors, confirming the performance in the literature, and posing an interesting prospect for future research.

## Simple Summary

Although neuroendocrine tumors are generally rare, they are the most common malignant neoplasm in the small intestine and the second most common gastrointestinal neuroendocrine location. Contrary to the historical conception of neuroendocrine neoplasms as indolent and non-aggressive, a significant percentage of cases present with lymph node or distant metastases at diagnosis. Nevertheless, they are significantly less studied in the literature than pancreatic neuroendocrine tumors. Histopathological evaluation still plays a crucial role in determining the prognosis and tailoring the treatment of patients with NETs. Radiomics is a quantitative analysis technique that enables the extraction and analysis of features imperceptible to the human eye from medical images with the aim of quantifying tumor imaging characteristics. In this study, we decided to investigate radiomic features extracted from CT images, focusing on small bowel NETs and evaluating their association with Ki-67 expression.

## 1. Introduction

Small bowel neuroendocrine tumors (SB-NETs) are considered rare, but they are the most common small bowel malignancy and make up approximately 17% of all diagnosed NETs. The small bowel is the second most common gastro–entero–pancreatic primary site of NETs after the rectum. Advances in diagnostic imaging have recently increased the number of incidentally discovered SB-NETs [1,2]. The median overall survival is 103 months, and the 5-year survival rate is 69%, although survival decreases with the extent of disease. The incidence of SB-NETs increases with age, with a median age at diagnosis of 66 years and a peak incidence at 80 years of age. Most small bowel carcinoid tumors originate in the terminal ileum [1,2].

Despite historical views that they are relatively indolent neoplasms, SB-NETs have the potential to develop loco-regional and/or distant metastases (especially to the liver). Lymph node metastases and liver metastases can be detected in up to 90% and 60–80% of patients, respectively [3,4,5].

Histopathological evaluation plays a crucial role in determining the prognosis and tailoring the treatment of patients with NETs. The 2019 World Health Organization (WHO) classification of tumors of the digestive system provides guidelines for grading SB-NETs: tumor grading is assessed based on the Ki-67 index or number of mitoses per 10 high-power fields (Grade 1 Ki-67 index < 2% or <2 mitoses per 10 HPF, Grade 2 Ki-67 index 3–20% or 2–20 mitoses per 10 HPF, Grade 3 Ki-67 index > 20% or >20 mitoses per 10 HPF) [4,5]. As in most neoplasms, prognosis has been demonstrated to be correlated with Ki67 expression [6,7].

Radiomics is a quantitative analysis technique that enables the extraction of features from medical images such as CT, MRI, and PET, with the aim of quantifying tumor imaging characteristics. Unlike traditional visual inspection, radiomics provides objective and reproducible data, contributing to a more accurate characterization of neoplasms [8,9,10]. Through the automated analysis of complex patterns—often imperceptible to the human eye—radiomics offers valuable insights for diagnosis, prognostic stratification, and treatment response prediction [11,12,13,14,15].

In recent years, radiomic studies regarding neuroendocrine tumors have seen a substantial growth in numbers (3 studies in 2017; 62 studies in 2025); they also shifted their main goal, from tumor detection to preoperative tumor grading [16,17].

Most of the radiomic studies found in the literature on gastro–entero–pancreatic NETs (GEP-NETs) focus on pancreatic NETs, while SB-NETs generally represent a small fraction of the cases in larger multiorgan series [7,11].

Only one study analyzed patients with ileal neuroendocrine tumors, but without focusing on grading, while predicting the risk of developing complications due to mesenteric masses using clinical criteria and radiomics [16].

In this study, we decided to investigate radiomic features extracted from CT images, focusing on SB-NETs and evaluating their association with Ki-67 expression, using 1% as a tailored threshold. We chose this threshold instead of the WHO 3% threshold (which differentiates G1 and G2 neoplasms) since the majority of SB-NETs are low-grade (up to 75% G1) [18], but a significant percentage have the tendency to develop local/regional and/or distant metastases. Therefore, there is an important prognostic heterogeneity in the “Ki67 < 3%” group of neoplasms, making the 1% a reliable prognostic tool for SB-NETs, as already underlined in the literature [7,8].

## 2. Materials and Methods

**Figure 1:**
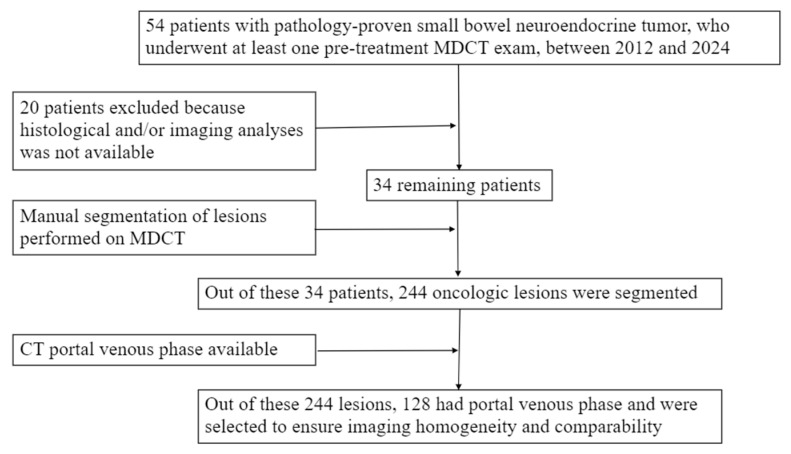
Lesion selection flow chart.

We initially selected 54 patients from two different institutes with pathology-proven small bowel neuroendocrine tumors, who underwent at least a pre-treatment contrast-media CT exam, in a period between 2012 and 2024. We excluded 20 patients because histological and/or imaging analyses were not available. Out of these 34 patients, 128 SB-NET lesions, acquired through computed tomography in portal phases, were selected (Figure 1). These lesions comprised primitive small bowel lesions, pathological lymph nodes or mesenteric tumor deposits, and secondary (liver) lesions that were divided as follows: 22 primitive lesions, 45 pathologic lymph nodes/mesenteric deposits, and 61 distant (liver) metastases. Patients and lesion characteristics are summarized in Table 1.

Each lesion was annotated with a binary label based on the Ki-67 proliferation index according to the following coding: class 0: Ki-67 ≤ 1%; class 1: Ki-67 > 1%. The dataset was moderately balanced, with 56 lesions in class 0 and 72 in class 1. The Ki67 proliferation index was assessed by two different pathologists in consensus; at least one pathologist had more than 10 years of experience in the evaluation of NETs.

We considered every segmented unit (primitive or secondary lesions) as an independent unit, extending the Ki67 value of primary lesions to pathological lymph nodes, mesenteric deposits, and secondary lesions if biopsy was not available. Primitive lesions were selected based on surgical and pathological reports, considering the location and size; pathological mesenteric lymph nodes and pathological mesenteric deposits were selected based on surgical and pathological reports or (if not available) based on imaging features (small axis ≥ 1 cm and peripheric fat stranding). Liver metastases were selected based on imaging features alone (since the majority of metastases were not biopsied).

**Figure 2:**
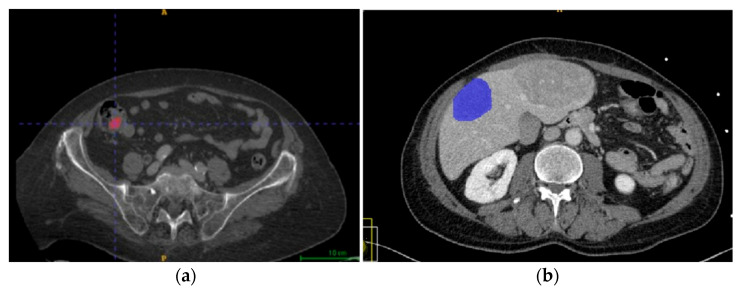
(**a**) Segmentation of the primary ileal NET in portal venous phase CT images, displayed in the axial plane. The segmented region of interest (ROI) corresponds to the primary ileal neoplasms and it is highlighted in red. (**b**) Segmentation of a liver metastasis from an ileal NET on portal venous phase CT images, displayed in the axial plane. The segmented ROI is highlighted in blue.

Segmentations of lesions were performed manually by one radiologist on portal-phase CT images using the segmentation program ITK-SNAP version 4.0 [13,19,20]; manual segmentation was performed to ensure the quality and accuracy of the region of interest (ROI) annotation, focusing only on small bowel NETs, primitive or secondary lesions with distinguishable margins, and free of significant artifacts. The segmentation was double-checked by an experienced radiology physician. Lesion size varied from ≈30 mm^3^ to ≈700 cm^3^, with a mean size of 100 cm^3^. Examples of segmented lesions are shown in Figure 2.

Medical imaging has been stored in DICOM format; CT images have been anonymized and centralized in one institute for radiomic analysis using secure transfer technology [13]. In order to ensure uniformity and quality, a range of pre-processing techniques has been applied by (using Python.org, https://www.python.org/), including intensity normalization, noise reduction, bias field correction, interpolation, and thresholding [13,21].

The open source PyRadiomics library (v3.0) was used for the radiomics extraction. It was implemented by instantiating the ‘featureextractor.RadiomicsFeatureExtractor()’ module, which allows for the extraction of radiomic features from regions of interest (ROI) in medical images. This algorithm systematically analyzes the ROIs, thereby enabling the quantification of a variety of morphological, statistical, and textural parameters. The features—calculated in compliance with the Image Biomarker Standardization Initiative (IBSI) guidelines to ensure reproducibility and comparability across studies—are typically classified into three categories: shape (geometry), first-order (intensity statistics), and texture (spatial patterns such as GLCM, GLRLM, GLSZM, NGTDM).

For each ROI, 107 radiomic features were extracted. The features were divided into 3 main categories: Shape features, first-order statistics, and texture features [22,23]. All variables were treated as continuous.

An initial exploratory analysis was performed on the radiomic features, including a distribution assessment, Spearman correlation matrix calculation, and removal of highly collinear variables (|*p*| > 0.95). Many variables demonstrated positive skewness, the presence of significant outliers, and leptokurtic distributions. Shape features, for instance, exhibited anisotropic geometries and asymmetrically distributed volumes. First-order features showed a wide range of intensity values, while texture features—including GLCM, GLRLM, GLDM, and GLSZM—highlighted marked patterns of internal heterogeneity. To identify statistically significant differences between the two classes, inferential statistical analysis was conducted through a Shapiro–Wilk test (for normality assessment), Levene’s test (for homogeneity of variances), *t*-test, and Mann–Whitney U test (respectively, parametric and non-parametric approaches, to evaluate the significance of differences—*p*-value < 0.05—in radiomic features between the two independent groups defined by the target variable, Ki-67 ≤ 1% vs. Ki-67 > 1%). For the final selection of features to be used in predictive modeling, four ranking methods were applied: Information Gain, Gini Decrease, ANOVA, and Chi-Squared test. The features scoring highly in at least three of the four methods were selected in accordance with the empirical “rule of thumb” that suggests using a number of features less than √N, where *p* is the number of selected features, and N is the total number of observations. This criterion aims to reduce overfitting and improve model generalizability [18]. Applying a correlation threshold of *p* < 0.05, 107 features were reduced to 41 nonhypercorrelated features.

An inferential analysis was performed on these remaining 41 features to identify those capable of discriminating between the two target classes. Nineteen features showed statistically significant differences (*p* < 0.05), based on either the Mann–Whitney U test or the independent *t*-test, depending on the distribution assessed using the Shapiro–Wilk and Levene’s tests.

The strongest instances of multicollinearity were observed among variables belonging to the same feature’s family. Nineteen features showed statistically significant differences between the two classes (*p* < 0.05). Eight features scoring highly in at least three of the four methods were selected in accordance with the empirical “rule of thumb” (details in Table 2).

A supervised learning approach was adopted to classify the lesions. Five classification models were implemented: Logistic Regression, Support Vector Machine (SVM), K-Nearest Neighbors (KNN), XGBoost, and Random Forest. Model training and validation were performed using 5-fold cross-validation (K = 5). In each iteration, the dataset was split into five subsets: four were used for training and one for testing, rotating across all combinations. To address class imbalance within training folds, the Synthetic Minority Oversampling Technique (SMOTE) was applied exclusively to the training set of each fold, resulting in balanced training subsets with 72 instances from each class (class 0 and class 1).

The adoption of K-Fold cross-validation and the use of SMOTE (Synthetic Minority Oversampling Technique) for class balancing ensured the robustness of our results. As also emphasized in the literature [21], such methodological strategies are essential when working with small and imbalanced datasets.

Hyperparameter tuning was performed via Grid Search, prior to model training, and was optimized based on accuracy. Model performance was evaluated using multiple metrics: Area Under the ROC Curve (AUC), which quantifies the classifier’s ability to distinguish between the two classes; Accuracy, the ratio of correct predictions to total observations; Precision, the proportion of correctly predicted positives, calculated as TP/(TP + FP); Recall (Sensitivity), the model’s ability to correctly identify positive cases, calculated as TP/(TP + FN); F1 score, the harmonic mean of Precision and Recall; Confusion Matrix, provides a detailed representation of predicted vs. actual classes, which is useful for analyzing classification errors by class.

## 3. Results

**Figure 3:**
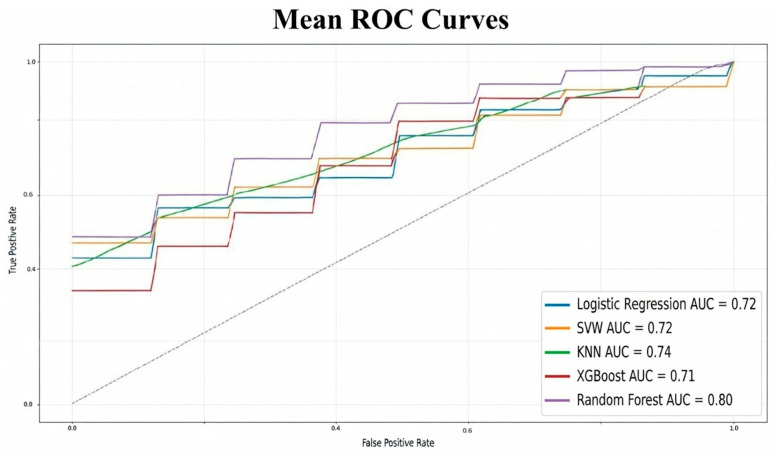
Mean ROC curves for all classifiers. Random Forest achieved the highest AUC (0.80), followed by KNN (0.74), Logistic Regression and SVM (both 0.72), and XGBoost (0.71). The dashed line represents the reference line for random classification (AUC = 0.5).

As shown in Figure 3 (mean ROC curves), the Random Forest model achieved the best performance, with a ROC AUC of 0.80, F1 score of 0.813, and recall of 0.847. K-Nearest Neighbors also performed well (AUC = 0.74), while Logistic Regression and SVM yielded intermediate results (AUC = 0.72). XGBoost exhibited higher variability, with an AUC of 0.71.

The aggregate performance metrics are summarized in Figure 3 and Table 3.

**Figure 4:**
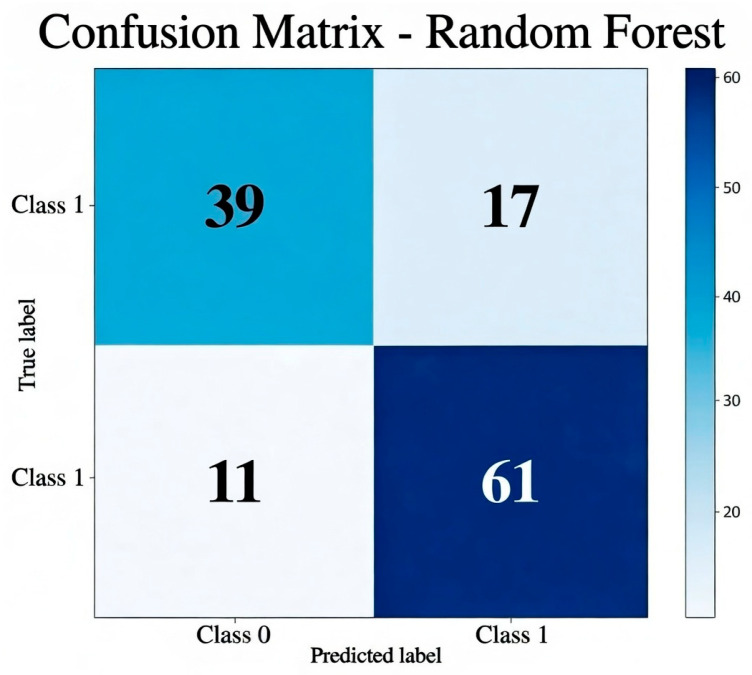
Confusion matrix of the best-performing model, Random Forest. The matrix shows classification outcomes for the two target classes, highlighting true positives, true negatives, false positives, and false negatives.

The confusion matrix for the Random Forest model (Figure 4) shows 61 true positives (class 1) and 39 true negatives (class 0), with 17 false positives and 11 false negatives. The main source of error is a false positive rate (FPR) of 30.4%. However, the low false negative rate (15.3%) is clinically relevant, as it reduces the risk of underestimating highly proliferative lesions.

## 4. Discussion

In this study, we investigated radiomic features extracted from CT images of small bowel neuroendocrine tumors and evaluated their association with Ki-67 index expression, posing a 1% threshold. As already explained, this Ki-67 cutoff, while different from the WHO grading system threshold of 3% that distinguishes G1 and G2, was chosen because the majority of SB-NETs are low-grade (up to 75% G1) [18], but a significant percentage have the tendency to develop local/regional and/or distant metastases. Therefore, there is a wide prognostic heterogeneity in the “Ki67 < 3%” group. The cutoff of 1% for Ki67 is consequently a reliable prognostic factor, as already highlighted in the literature by Klöppel et al. [8].

Several studies in recent years investigated the contribution of radiomics to neuroendocrine tumors detection, differential diagnosis, and staging, particularly through the analysis of contrast-enhanced CT or PET/CT images [17]. Most of the studies, though, have focused on pancreatic neuroendocrine tumors; this might be due to the overall higher incidence of pancreatic cancer compared to small bowel cancer (considering every histotype), even though neuroendocrine tumors demonstrate a similar incidence in the pancreas and small bowel (approximately 1–4/100,000/year) [5], and neuroendocrine neoplasms are the most frequent malignancy in the small bowel. In a 2022 literature review, 45 studies on gastro–entero–pancreatic NET radiomics were analyzed: most of these studies focused on predicting tumor grade and differential diagnosis between neuroendocrine tumors and other histotypes. Twenty-five studies constructed models to predict tumor grade, although they considered the WHO thresholds of the Ki67 index for grading (<3%, 3–20%, >20%), and used either radiomics-only models or combined clinical/radiological models, with AUC values ranging from 0.68 to 0.90 [11,21,22,24,25].

In this study, we selected the most significant eight radiomic features, three of which are first-order features (firstorder_RootMeanSquared, firstorder_Maximum, firstorder_10percentile), i.e., quantitative measures that characterize the spatial relationship and distribution of voxel intensities, providing a numerical representation of the image’s texture and heterogeneity. First-order radiomic features have been predominantly studied in the literature, although with conflicting results; in general, an increased heterogeneity (higher entropy, kurtosis, max intensity, and lower energy) has been found to be associated with higher-grade tumors [26,27,28,29]. The other five features are second-order or texture features (statistical measures that describe the distribution of voxel intensity values within a ROI without considering the spatial relationships between voxels): glszm_GrayLevelNonUniformity, glszm_ZoneEntropy, glcm_Correlation, glszm_SmallAreaEmphasis, ngtdmStrenght). Second- or higher-order features have also been studied, to find application in the histologic characterization of pancreatic neuroendocrine tumors: in 2021, Benedetti et al. [17] identified six second- or higher-order features that correlate with vascular invasion, with AUCs ranging between 0.77 and 0.81. For our study’s analysis, we only implemented Machine Learning algorithms (Logistic Regression, SVM, KNN, XGBoost, Random Forest); a detailed analysis of model performance revealed Random Forest as the best-performing classifier, achieving an average ROC AUC of 0.80 and a recall of 0.847. This might be significant from a clinical perspective: a low false negative rate reduces the risk of underestimating more aggressive tumors, facilitating a more cautious follow-up and a timely therapeutic approach. Machine Learning algorithms were also largely implemented in the literature, probably because of the paucity of data: deep learning is based on artificial neural networks with multiple hidden layers, and because of their deep architecture and the vast number of parameters to optimize, these models need a massive amount of data to prevent overfitting and to generalize effectively. Without enough data, the network cannot learn the complex patterns and hierarchical representations that make it powerful. Furthermore, results based on deep learning are controversial; Luo et al. compared machine learning and deep learning algorithms’ performances to predict pancreatic neuroendocrine neoplasms grading based on CT imaging and found that a deep learning model can predict tumor grade, yet not significantly better than a traditional ML radiomics model [28]. Liang investigated tumor grade prediction in rectal NET through radiomics (CT histogram analysis) [27]. In the only other study in the literature focused on SB-NETs, Anela Blazevic et al. showed that a radiomic-based model can predict the risk of complications in patients with mesenteric masses in small bowel NETs in 68 patients (32 in the asymptomatic group and 36 in the symptomatic group) with an AUC of 0.81 (sensitivity 0.78 and specificity 0.67), which is very similar to our accuracy [16].

Although promising, this study has several limitations. The limited number of datasets (patients and lesions) affects the generalizability of the findings. This limit is mostly due to the rarity of the neoplasm, and it may be overcome by amplifying the number of research centers participating.

Due to the paucity of lesions, we have not been able to implement a Deep Learning algorithm, which requires datasets (tens/hundreds of thousands of independent units) to implement the neural links, and we had to implement classic Machine Learning models in order to avoid overfitting; this limitation is shared with most of the previous literature on the subject.

The use of a tailored cutoff for Ki-67 (1% instead of the WHO 3%) may reduce the direct comparability of results to the studies that used the standard WHO classification value. As already mentioned, though, we chose this cutoff because the majority of SB-NETs are low-grade, but a significant percentage are not indolent, making the 1% valuable for prognosis, as highlighted in the literature [7,8].

We also generalized the Ki67 index expression value, extending it from the primary tumor to the secondary lesions, even when not biopsied—a technique that is often used in the literature (but not ideal) in clinical practice.

Another limitation is the retrospective design; in this study, we selected CT exams from two different institutes and acquired them in different CT scans and in different settings (some of which came from the ER setting); therefore, acquisition and reconstruction parameters and the type of contrast media may present substantial differences.

Due to the limited number of patients and lesions, only internal cross-validation was possible at this stage. We emphasize that external validation on independent datasets will be conducted as soon as a larger number of cases and CT images are available.

Integrating multiparametric data, including genomics and clinical variables, may also significantly boost the predictive power of radiomic models, and it is a future research prospect for this subject.

## 5. Conclusions

As radiomics advances in every field of radiology, this study highlights its potential for non-invasive assessment of proliferative rate of small bowel neuroendocrine tumors, confirming the performance in the literature, and posing an interesting prospect for future research.

## Figures and Tables

**Table 1:** Patients and lesions statistics.

	Ki67 ≤ 1%	Ki67 > 1%
**Number of Patients**	19	15
**Mean age at diagnosis**	62.2	69.4
**Sex**	M: 11, F: 8	M: 8, F: 7
**Tumor Grade**	G1: 19	G1: 5, G2: 10, G3: 0
**Number of independent Lesions**	56 (11 primary tumors, 23 lymph nodes/mesenteric deposits, 22 metastases)	72 (11 primary tumors, 22 lymph nodes/mesenteric deposits, 39 metastases)

**Table 2:** Radiomic features ranking based on multiple scoring criteria (Information Gain, Gini, ANOVA, and χ^2^). Eight features (#1–8) scoring highly in at least three of the four methods were selected in accordance with the empirical “rule of thumb”.

#	Feature	Info. Gain	Gini	ANOVA	X
**1**	**original_firstorder_RootMeanSquared**	**147**	**89**	**18,332**	**16,593**
**2**	**original_firstorder_Maximum**	**108**	**71**	**4978**	**13,758**
**3**	**original_glszm_GrayLevelNonUniformity**	**99**	**65**	**4603**	**13,228**
**4**	**original_glszm_ZoneEntropy**	**94**	**60**	**10,878**	**6116**
**5**	**original_firstorder_10Percentile**	**75**	**49**	**6867**	**10,718**
**6**	**original_glcm_Correlation**	**75**	**48**	**9283**	**7640**
**7**	**original_glszm_SmallAreaEmphasis**	**73**	**47**	**5016**	**5418**
**8**	**original_ngtdm_Strength**	**73**	**48**	**64**	**6116**
9	original_glrlm_RunEntropy	69	44	9050	6519
10	original_ngtdm_Busyness	68	46	9900	8466
11	original_glszm_ZoneVariance	68	46	3384	8466
12	original_glszm_LargeAreaLowGrayLevelEmphasis	68	46	8497	6116
13	original_ngtdm_Coarseness	66	44	69	9333
14	original_glcm_InverseVariance	64	42	5166	7640
15	original_glrlm_LongRunHighGrayLevelEmphasis	62	42	14	5418
16	original_glcm_MCC	56	36	7558	6116
17	original_shape_Sphericity	40	26	5124	4762
18	original_glszm_SizeZoneNonUniformity	33	22	743	3577
19	original_gldm_SmallDependenceLowGrayLevelEmphasis	26	18	2561	3084

**Table 3:** Aggregated classification metrics for each Machine Learning (ML) predictive model.

Model	ROC AUC	Accuracy	F1 Score	Precision	Recall
**Logistic regression**	0.72 ± 0.02	0.734 ± 0.015	0.767 ± 0.015	0.757 ± 0.018	0.778 ± 0.017
SVM *	0.72 ± 0.02	0.734 ± 0.017	0.761 ± 0.017	0.771 ± 0.016	0.750 ± 0.020
**KNN ^#^**	0.74 ± 0.03	0.719 ± 0.020	0.746 ± 0.025	0.757 ± 0.023	0.736 ± 0.021
**XGBoost**	0.71 ± 0.02	0.727 ± 0.018	0.774 ± 0.015	0.723 ± 0.020	0.833 ± 0.018
**Random Forest**	0.80 ± 0.01	0.781 ± 0.015	0.813 ± 0.012	0.782 ± 0.014	0.847 ± 0.013

* SVM: Support Vector Machine; ^#^ KNN: K-Nearest Neighbors.

## Data Availability

The data presented in this study are available on request from the corresponding author due to privacy reasons.

## Abbreviations

The following abbreviations are used in this manuscript:

SB-NETs	Small Bowel Neuroendocrine Tumors
GEP-NETs	Gastro–entero–pancreatic NETs
ML	Machine Learning
SVM	Support Vector Machine
KNN	K-Nearest Neighbors
